# QC*omics*: Recommendations and Guidelines for Robust, Easily Implementable and Reportable Quality Control of Metabolomics Data

**DOI:** 10.1021/acs.analchem.3c03660

**Published:** 2024-01-05

**Authors:** Álvaro González-Domínguez, Núria Estanyol-Torres, Carl Brunius, Rikard Landberg, Raúl González-Domínguez

**Affiliations:** † Instituto de Investigación e Innovación Biomédica de Cádiz (INiBICA), Hospital Universitario Puerta del Mar, Universidad de Cádiz, Cádiz 11009, Spain; ‡ Division of Food and Nutrition Science, Department of Life Sciences, Chalmers University of Technology,SE-412 96Gothenburg ,Sweden

## Abstract

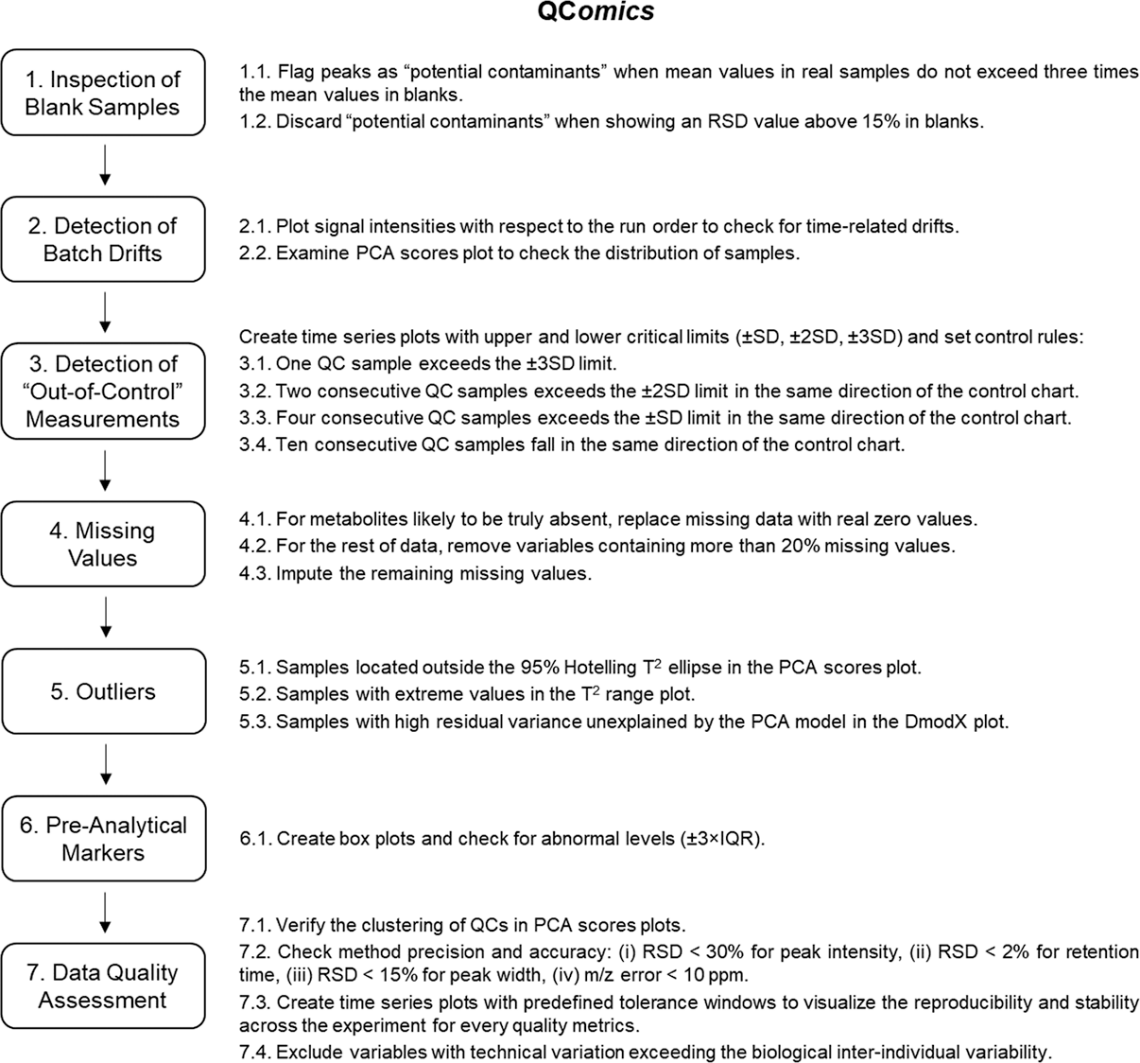

The implementation of quality control strategies is crucial to ensure the reproducibility, accuracy, and meaningfulness of metabolomics data. However, this pivotal step is often overlooked within the metabolomics workflow and frequently relies on the use of nonstandardized and poorly reported protocols. To address current limitations in this respect, we have developed QC*omics*, a robust, easily implementable and reportable method for monitoring and controlling data quality. The protocol operates in various sequential steps aimed to (i) correct for background noise and carryover, (ii) detect signal drifts and “out-of-control” observations, (iii) deal with missing data, (iv) remove outliers, (v) monitor quality markers to identify samples affected by improper collection, preprocessing, or storage, and (vi) assess overall data quality in terms of precision and accuracy. Notably, this tool considers important issues often neglected along quality control, such as the need of separately handling missing values and truly absent data to avoid losing relevant biological information, as well as the large impact that preanalytical factors may elicit on metabolomics results. Altogether, the guidelines compiled in QC*omics* might contribute to establishing gold standard recommendations and best practices for quality control within the metabolomics community.

## Introduction

The metabolome encompasses a multitude of metabolites with diverse physicochemical properties, including substrates and end-products that participate in the endogenous metabolism, metabolites derived from absorption and biotransformation of exogenous compounds from diet, lifestyle habits, and environmental pollution (i.e., the exposome), and microbiota-related metabolites.^1,2^ Accordingly, mass spectrometry (MS)-based metabolomics generates vast and complex data, comprising hundreds to thousands of molecular features that exhibit wide concentration ranges and large interindividual variability. The acquisition of reproducible and meaningful data requires the application of standard operating procedures (SOPs) to minimize human errors (e.g., errors in pipetting during sample processing), random errors (e.g., fluctuations allocated to intrinsic method precision and other analytical limitations), and systematic errors (e.g., biases that persist throughout the analytical process). However, unwanted sources of variation are hardly controllable and make the implementation of quality control (QC) strategies mandatory to monitor and control data quality along the entire analytical workflow.^3−5^ On the one hand, metabolite levels can be influenced by a myriad of preanalytical factors related to sample collection and preprocessing, conditions that must be tightly controlled to guarantee the metabolic integrity of biological samples under study.^6^ Moreover, although metabolomics normally employs straightforward extraction protocols, the chemical complexity of biological matrices requires, at least, the removal of proteins and potential interferences (e.g., salts and lipids) and other additional method-specific steps (e.g., preconcentration and derivatization).^7^ Despite numerous efforts made for its automation, sample preparation still largely relies on human handling in many laboratories, thus being considered to be one of the most error-prone steps in metabolomics. Finally, instrumental stability issues and fluctuations in analytical performance also have a negative impact on reproducibility and accuracy, especially when dealing with large-scale epidemiological studies or complex samples. Along the experiment, the MS system may suffer from significant drifts in sensitivity, mass accuracy, retention time (RT), and peak resolution due to different reasons: (i) contamination with components from the matrix (e.g., lipids), mobile phase (e.g., buffers), or other impurities (e.g., plasticizers); (ii) deterioration and clogging of chromatographic columns; (iii) uncontrolled temperature in the laboratory and/or instrument components (e.g., autosampler trays), which may provoke degradation and precipitation of samples and mobile phases, as well as to strongly influence the performance of separation, ionization, and detection processes.^4^ In this context, several scientific task-force groups have been established to address current challenges and provide general recommendations for proper QC among the metabolomics community, such as the Metabolomics Society Data Quality Task Group (DQTG)^8,9^ and the metabolomics Quality Assurance and quality Control Consortium (mQACC).^10−12^ However, despite these efforts, only a few standardized protocols have been published up to date with the aim of harmonizing this crucial QC step within the metabolomics workflow.^13−16^

In conventional targeted analysis, variability factors are typically addressed by using spiked internal standards (ISs), most often consisting of isotopically labeled compounds. However, this approach is not viable in untargeted metabolomics as samples may contain thousands of a priori unknown metabolites. A compromise solution commonly applied in large-scale targeted metabolomics is to use a set of ISs representing the physicochemical space of the method coverage (i.e., at least one IS per metabolite class).^2,17^ As an alternative, the analysis of biological QC samples is nowadays the gold standard in metabolomics, as first proposed by Sangster et al. in 2006.^13^ These QC samples are usually prepared by pooling equal volumes of all samples under investigation, although surrogate QCs (e.g., commercially available biological samples or certified reference materials) can also be employed when the pooling strategy is not practicable (e.g., large epidemiological cohorts). Then, QC samples are analyzed at the beginning of the analytical run to equilibrate the instrument, as well as at intermittent points throughout the experiment to monitor system stability and correct experimental variability sources during data processing.^18,19^ Furthermore, some authors have proposed the use of serially diluted QC samples to discard molecular features lacking a linear correlation between the MS response and the relative concentration, which are expected to be artifacts rather than true biological signals.^20^ However, it should be noted that correlation analysis is inherently sensitive to the dilution strategy (e.g., inclusion of diluted QCs at very high/low concentrations). As metabolites can be present in very different and wide concentration ranges, this strategy should consider enough dilution points to properly address linearity, thereby considerably increasing analysis times and hindering subsequent data processing. After analysis, to assess method reproducibility, most QC approaches solely rely on (i) inspecting the clustering of QCs in principal component analysis (PCA) scores plots and (ii) computing the relative standard deviation (RSD) for metabolites of interest across QC samples.^13−16^ The main limitation of these previously published QC protocols is their subjective nature and lack of harmonization between laboratories, since predefined quality criteria and standard procedures have not yet been established, as recently highlighted by the mQACC.^11^ Furthermore, it should be noted that these methods mainly focus on addressing analytical variability (e.g., drifts along the sequence run) but frequently underestimate the great impact that preanalytical factors may elicit on metabolomics results. Considering the growing interest in the exposome and its relationship with health outcomes, it also becomes essential to adapt current QC practices for dealing with heterogeneous data sets comprising both endogenous metabolites and xenobiotics, which are normally present at very different concentration levels in the organism.

Herein, we present “QC*omics*”, a comprehensive protocol for QC assessment of metabolomics data based on a sequential multistep workflow: (i) initial data exploration (i.e., detection of contaminants, batch drifts, and out-of-control measurements), (ii) handling missing values and truly absent data, (iii) removal of outlying samples, (iv) monitoring quality markers to address preanalytical errors, and (v) final data quality assessment. The quality criteria employed in QC*omics* have been adapted from guidelines for validating conventional bioanalytical methods^21^ and from existing literature on current QC practices in metabolomics.^3,11^ To simplify its implementation, this QC protocol can easily be performed in software that is available to most researchers (e.g., Microsoft Excel and MetaboAnalyst webtool), without the need of advanced statistical and programming skills.

## Experimental Section

### Blank and Quality Control Samples

The implementation of QC*omics* requires procedural blanks and QC samples, obtained as follows. Blank samples must be prepared by replacing the biological sample under study with water during the extraction process but using the same chemicals, labware, and SOPs as for real samples. In the case of simple extraction protocols (e.g., protein precipitation with organic solvents), blank extraction solvents can instead be employed for this purpose. On the other hand, the QC sample is prepared by mixing equal aliquots of each of the samples under investigation or by using a bulk representative sample when the pooling strategy is not viable. Before analysis, the QC sample must be treated by applying the same extraction procedure used for real samples. Optionally, study samples can also be spiked with a set of ISs, as traditionally done in targeted analysis.

### Metabolomics Analysis

To develop and validate QC*omics*, we leveraged metabolomics data that were generated using the untargeted approach described by González-Domínguez et al. as a case study.^22^ Briefly, plasma samples were treated with cold acetonitrile for protein precipitation, and metabolite extracts were then analyzed by reversed-phase ultrahigh-performance liquid chromatography coupled to high-resolution mass spectrometry (UHPLC-MS), using the operating conditions described elsewhere.^22^ The injection order of samples in the MS system should follow this sequence:

(1) Inject five consecutive procedural blank samples to stabilize the system (e.g., operating temperatures and chromatographic pressure) and to check the background noise.
(2) Inject several consecutive QC samples to condition the system for the study matrix (i.e., stable chromatographic pressure, reproducible RT, peak area, and peak shape for selected metabolites). This conditioning step usually requires at least five QC samples, although this number might be increased (e.g., 10 injections) when studying complex matrices (e.g., tissues) and when applying less robust analytical approaches (e.g., hydrophilic interaction liquid chromatography, HILIC).
(3) Analyze real samples in random order and intercalate QCs across the sequence (e.g., one QC after every 10 samples). If the sample size is small, the frequency of QC injection may be increased to ensure a minimum of 10% QC samples across the analytical run.
(4) Inject five procedural blank samples at the end of the sequence run to assess carryover. We do not recommend intercalating blank samples throughout the sequence as this may result in partial deconditioning of the system (e.g., shifts in RT and peak symmetry) due to differences in matrix composition, which would make necessary injecting several reconditioning QCs after blanks before continuing with the analysis of real samples, thereby lengthening total run times.

After MS-based analysis, a set of metabolites that can regularly be detected in QC samples (hereinafter referred to as “chemical descriptors”) must be selected to assess method reproducibility and data quality. These metabolites should preferably belong to different chemical classes representing the analytical coverage of the MS method, have diverse molecular weights (MW) and peak intensities, and be well-distributed along the chromatographic run. In targeted experiments, spiked ISs can be used as additional quality markers. Herein, for the particular case of reversed-phase UHPLC-MS analysis of plasma samples, we propose the set of chemical descriptors listed in Table 1. Note that *m*/*z* and RT values correspond to those obtained by applying the metabolomics method described elsewhere,^22^ the user should adapt the set of chemical descriptors according to the analytical performance of their own methods.

**Table 1:** Set of Chemical Descriptors to Assess the Method Reproducibility and Data Quality

**metabolite**	**monoisotopic mass (Da)**	*m*/*z***(Da)**	**retention time (min)**
creatinine	113.0589	[M + H]^+^: 114.0667	0.68
		[M – H]^−^: 112.0511	
l-phenylalanine	165.0790	[M + H]^+^: 166.0868	2.01
		[M – H]^−^: 164.0712	
hippuric acid	179.0582	[M + H]^+^: 180.0660	3.02
		[M – H]^−^: 178.0504	
indole-3-acetic acid	175.0633	[M + H]^+^: 176.0711	3.98
		[M – H]^−^: 174.0555	
cortisone	360.1937	[M + H]^+^: 361.2015	4.84
		[M + Cl]^−^: 395.1626	
dehydroepiandrosterone 3-sulfate	368.1657	[M + Na]^+^: 391.1555	5.36
		[M – H]^−^: 367.1579	
cholic acid	408.2876	[M + Na]^+^: 431.2774	6.30
		[M – H]^−^: 407.2798	
stearoyl-L-carnitine	427.3656	[M + H]^+^: 428.3734	6.45
monooleoyl-glycerol	356.2927	[M + Na]^+^: 379.2825	7.07
palmitic acid	256.2402	[M + Na]^+^: 279.2300	7.22
		[M – H]^−^: 255.2324	
bilirubin	584.2635	[M + H]^+^: 585.2713	7.78
		[M – H]^−^: 583.2557	
1,2-dipalmitoyl-phosphatidylcholine	733.5622	[M + Na]^+^: 756.5520	9.19
		[M + Cl]^−^: 768.5311	

### Implementation of QC*omics*

**Figure 1:**
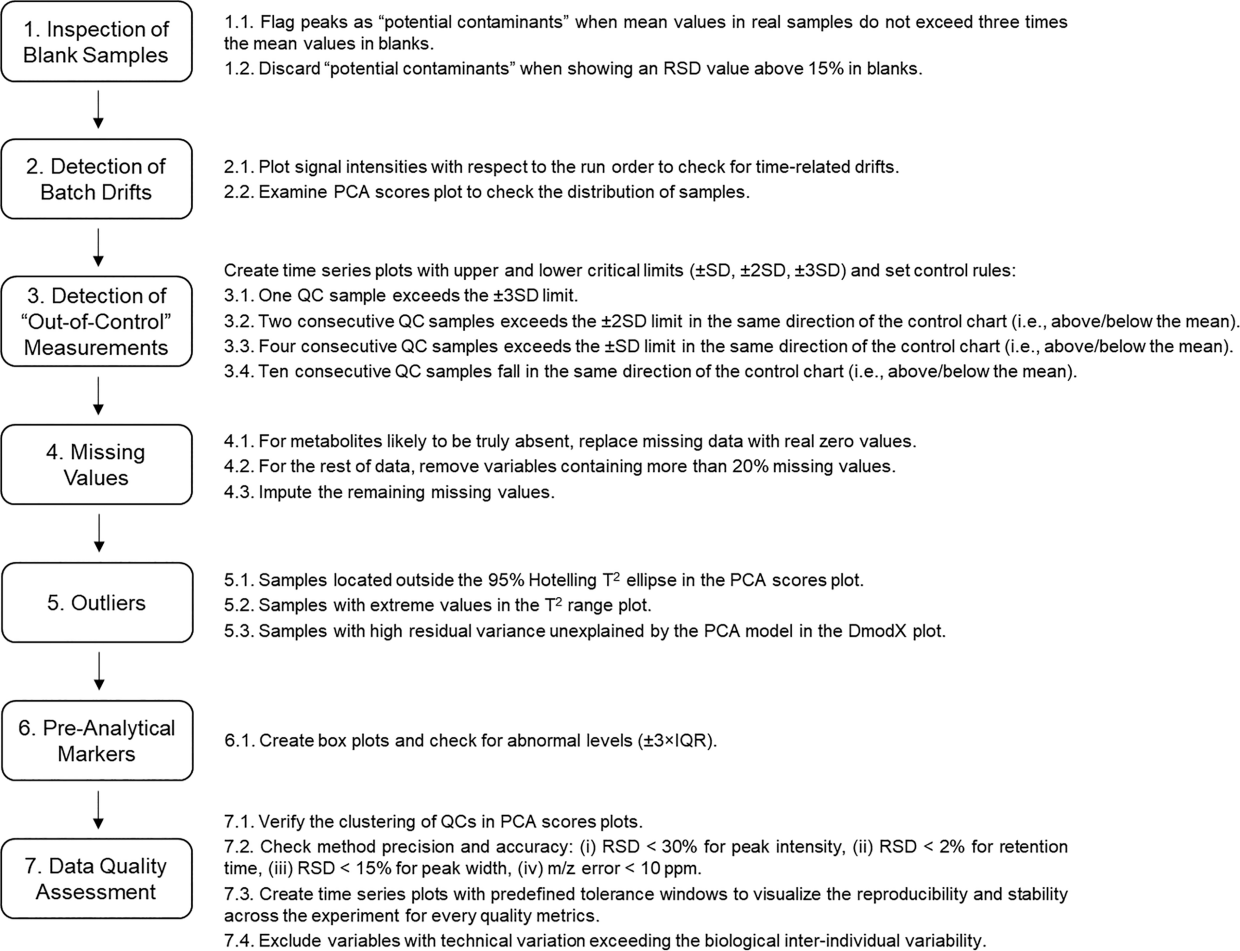
Overview of the QC*omics* workflow.

The QC*omics* protocol operates through a multistep workflow to sequentially address various challenges that may strongly influence data quality, as detailed in Figure 1. In the next sections of this article, we discuss recommendations and guidelines for properly dealing with background noise and potential contaminants, batch drifts and “out-of-control” measurements, missing values and truly absent data, outlying samples, improper handling/storage of biological samples, and overall quality assessment.

## Results and Discussion

### Initial Data Exploration

The first step in QC*omics* involves a preliminary exploratory data analysis to check for potential contaminants, carryover, trends according to the run order, and “out-of-control” measurements.

#### Inspection of Procedural Blank Samples

Metabolomics data, especially when applying untargeted approaches designed to detect as many molecular features as possible, are prone to contain artifact signals originating from sources other than the biological matrix under investigation, such as additives and preservatives incorporated during sample collection and processing, ghost peaks derived from sample preparation (e.g., derivatization), impurities present in solvents and reagents, and contaminants coming from labware and the MS system (e.g., plasticizers or column bleeding). Furthermore, these contaminants and other matrix components may accumulate in the instrument (e.g., in the autosampler or in the column) as a result of inadequate washing between sample injections, leading to the appearance of carryover signals from the foregoing samples in subsequent injections.

To minimize the impact of this background noise, procedural blank samples must be injected at the beginning and at the end of the sequence run to identify potential artifacts in the data set.^23^ As the exclusion criterion, QC*omics* flags molecular features as a “potential contaminant” when their mean values in real samples do not exceed three times the mean values detected in blanks, as recently reported by the mQACC.^11^ However, we recommend not removing these peaks prior to data analysis as some of them can be biologically relevant (e.g., free fatty acids that are frequently employed as slip agents in plastic consumables). Instead, if the “contaminant” is selected as of potential interest after data analysis, the researcher should determine to which extent the blank contribution might influence the quality of results. To this end, we propose discarding only those features with an RSD value higher than 15% in blank injections, as low blank-related variability is not expected to differentially affect group comparisons or statistical testing.

#### Detection of Batch Drifts and “Out-of-Control” Measurements

**Figure 2:**
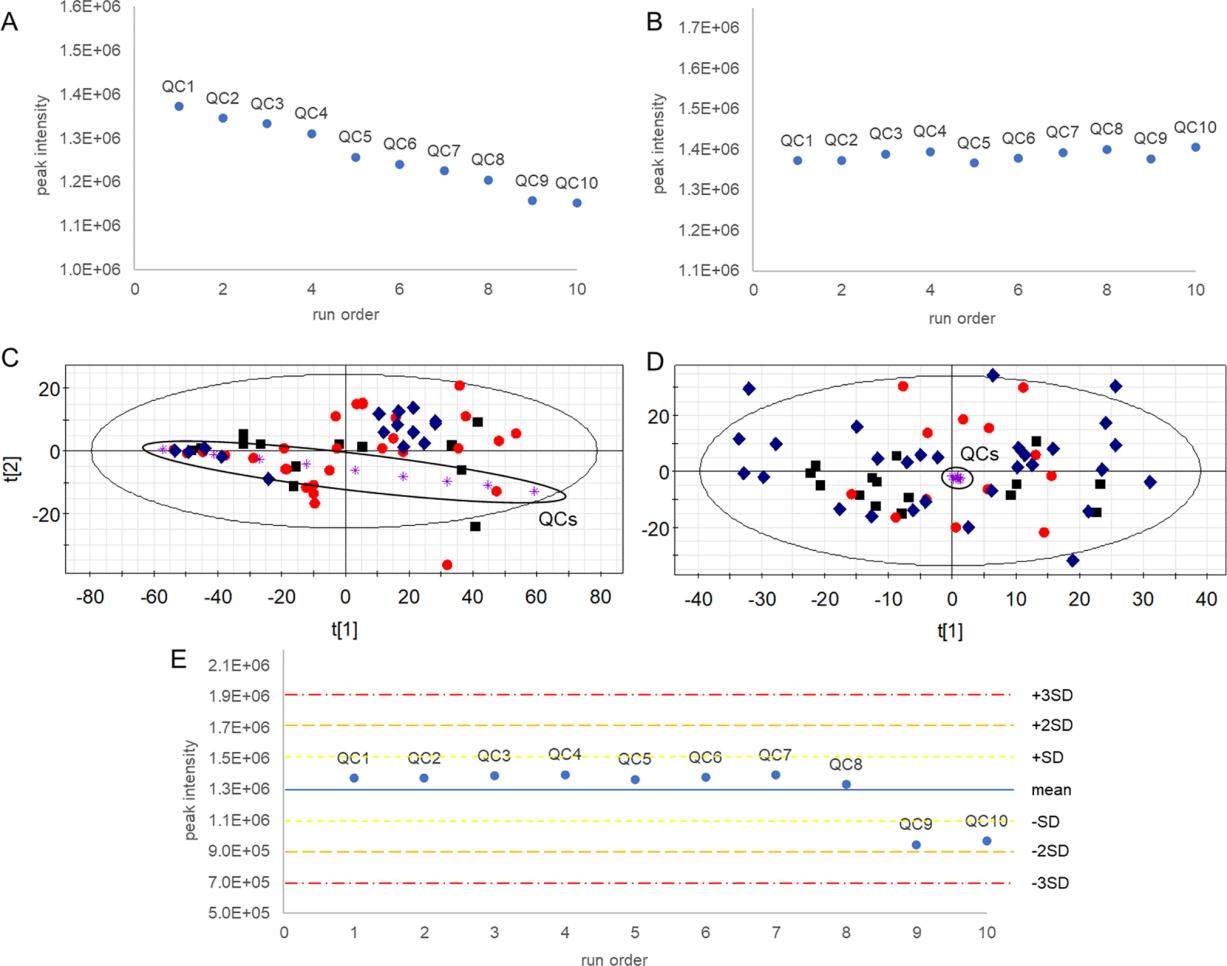
Detection of batch drifts and “out-of-control” measurements. (A) Time series plot showing signal drift in quality control samples; (B) time series plot showing stable signal in quality control samples; (C) principal component analysis scores plot showing signal drift in quality control samples; (D) principal component analysis scores plot showing stable signal in quality control samples; (E) Shewhart control chart for detecting “out-of-control” measurements.

The most frequent origin of systematic errors in metabolomics is deficient instrumental stability (e.g., loss in MS sensitivity or deterioration of chromatographic columns), which is ultimately mirrored in signal drifts according to the run order. To verify the presence of gradual changes along the analytical run, and thus evaluating the need of implementing data normalization approaches,^19^ the peak intensity of each chemical descriptor should be plotted with respect to their run order to check for time-related trends in the data (Figure 2A,B). Then, the examination of PCA scores plots enables exploring if samples show a continuous drift (Figure 2C) or a homogeneous distribution (Figure 2D) in the PCA space. If prepared by pooling, QC samples should ideally cluster in the center of the plot.

Besides the abovementioned gradual drifts in signal, the analytical system can also experience sudden deterioration (e.g., column clogging), thereby resulting in “out-of-control” measurements that are hard to correct through normalization approaches. In that case, the implementation of Shewhart control charts would facilitate the detection of abnormal QC samples to determine if a batch is acceptable or not (Figure 2E). To detect “out-of-control” measurements, create time series plots with upper and lower critical limits (±SD, ± 2SD, ± 3SD) for each chemical descriptor and set the control rules proposed by Westgard et al.:^24^ (i) one QC sample exceeds the ±3SD limit; (ii) two consecutive QC samples exceed the ±2SD limit in the same direction of the control chart (i.e., above or below the mean); (iii) four consecutive QC samples exceed the ± SD limit in the same direction of the control chart (i.e., above or below the mean); (iv) 10 consecutive QC samples fall in the same direction of the control chart (i.e., above or below the mean). If QC samples do not meet these quality criteria, neighboring study samples should be scrutinized to evaluate the necessity of being discarded and reanalyzed.

### Handling Missing Values and Truly Absent Data

Metabolomics data sets are typically characterized by high frequency of missing values (ca. 20–30% of overall data), which poses additional challenges during QC assessment. The origin of missing values in MS-based metabolomics can be allocated to a myriad of instrumental reasons, including sensitivity limitations (i.e., metabolite levels below the analytical limit of detection), technical issues (e.g., matrix effects or coelution), and random errors (e.g., temporary reduction in ionization performance). This results in diverse types of missing values, including missing completely at random (MCAR, i.e., missing data is independent of observed and unobserved data), missing at random (MAR, i.e., missing data is dependent on observed data), and missing not at random (MNAR, i.e., missing data is dependent on unobserved data). Moreover, missing data can also arise from the true absence due to biological reasons (e.g., xenobiotics that are exclusively detected in exposed individuals). Nevertheless, common strategies for dealing with missing values in metabolomics do not account for this heterogeneity and simply rely on a two-step process for filtering variables containing a high proportion of missing values and subsequent imputation of remaining data.^25,26^ This may provoke the loss of relevant biological information during the filtering step (e.g., exogenous metabolites with low detection rate) and lead to inaccurate and biased results due to suboptimal imputation.

The QC*omics* tool comprises a novel protocol for differentially addressing missing and truly absent values, of particular interest in exposomics and nutritional metabolomics. First, we compute the rate of missing values per study sample to confirm a consistent distribution along the entire analytical run and to discard data with abnormally lower detection rates. This and further steps should be performed separately in each study group to account for group-specific metabolite occurrences (e.g., xenobiotics coming from an intervention trial). Data must then be scrutinized with the aim of distinguishing missing values from potentially truly absent variables to differentially treat them in subsequent steps. For this purpose, we assume that a low proportion of missing values in any variable is likely to indicate false absence due to analytical issues, while high frequency of missing values could be allocated to true absence, as previously reported by Armitage et al.^27^ Using synthetic data sets, they found that the proportion of missing values strongly affects subsequent statistical testing, so that the true-positive rate was drastically reduced as the number of missing values increased, but it was rapidly restored with more than 70% missing values. In that scenario, a compromise between true presence and true absence might be parametrized based on the information that is not lost (i.e., false negatives) nor gained (i.e., false positives) during the imputation process. Based on this rationale, it is proposed that molecular features with more than 70% missing values could actually be regarded as metabolites likely to contain real zero values. This categorization is even simpler in targeted metabolomics, where the identities of analytes are a priori known. In that particular case, we assume that missing values in endogenous metabolites may have a plausible technical origin, as they are expected to be regularly detected in all samples analyzed. In contrast, missing values in exogenous metabolites (e.g., dietary compounds, drugs, or pollutants) could be regarded as truly absent data. Once this categorization is accomplished, QC*omics* handles missing values in various steps. For metabolites likely to be truly absent (i.e., molecular features with >70% missing values in untargeted metabolomics, exogenous metabolites in targeted metabolomics), missing data are replaced with real zero values. In such cases, special care must be taken during subsequent statistical analyses to properly deal with zero-inflated data. For the rest of the data, variables containing more than 20% missing values in all the study groups should be removed to discard spurious signals. Finally, the remaining missing values are imputed by using the method of choice (e.g., kNN or Random Forest). Although rather simplistic, as the unequivocal differentiation between missing values and absent data is difficult in practice, this categorization-based approach could represent a complementary alternative to traditional imputation strategies,^25,26^ especially with the aim to get deeper insights into the role of the exposome in health status. This is of particular interest when studying pollutants and toxicants, which are usually present at extremely low concentrations in biological specimens and are hardly detectable using metabolomics approaches. In that case, traditional imputation methods based on filtering variables that contain high proportions of missing values (e.g., 80% rule) are expected to remove most exposome-related features from metabolomics data sets, while keeping these missing data as real zero values would prevent losing relevant information.

### Detection and Removal of Outliers

**Figure 3:**
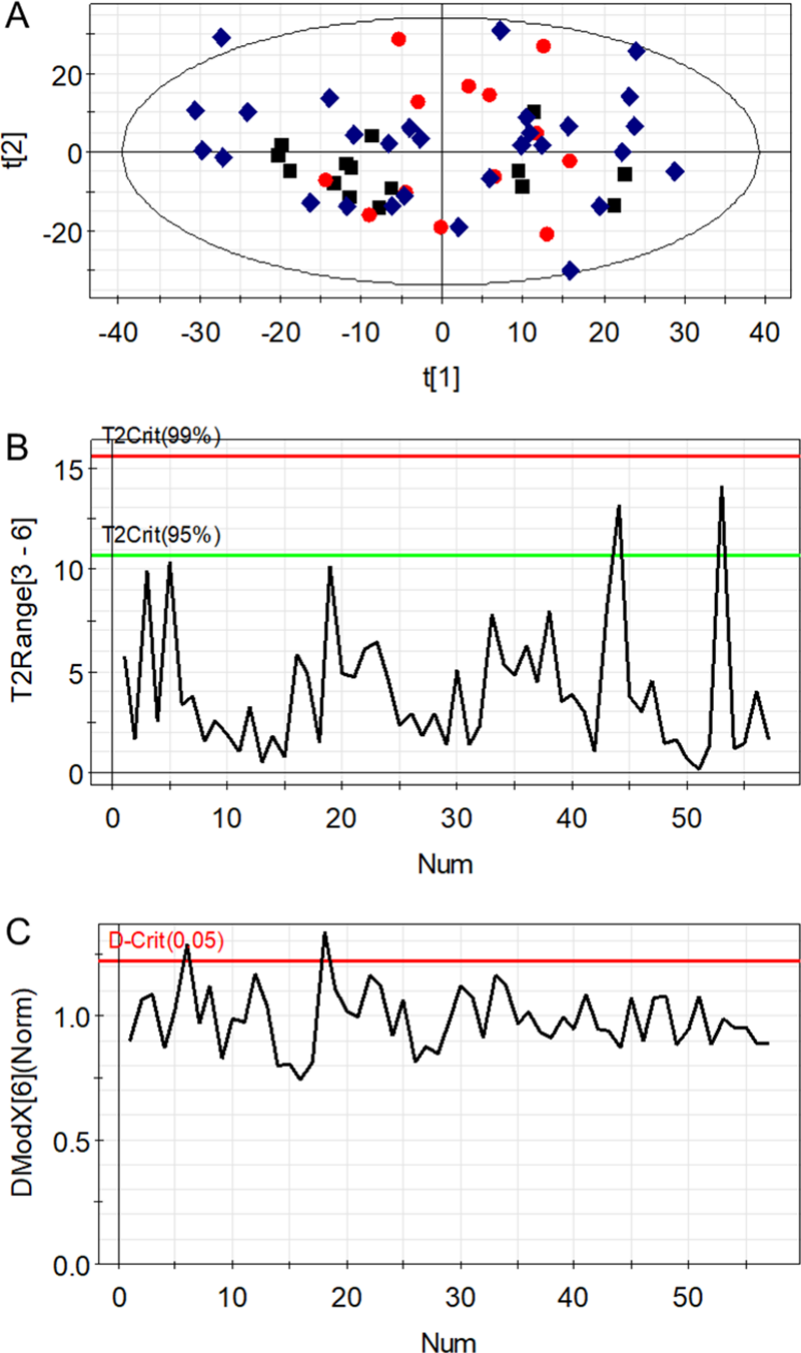
Detection of outliers. (A) Principal component analysis scores plot with 95% Hotelling T^2^ ellipse; (B) T^2^ range plot; (C) DmodX plot.

The detection and removal of outliers (i.e., data points that significantly deviate from the remaining observations) are crucial steps in MS-based metabolomics as they can originate from multiple sources of analytical and biological variability, consequently leading to inaccurate results. A great number of methods have been proposed for these purposes based on the Mahalanobis distance,^28^ Grubbs’s test,^29^ and nonlinear regression.^30^ Among them, PCA and the Hotelling T^2^ test have emerged as the gold standard.^31^ As implemented in QC*omics*, the analysis of PCA scores using the Hotelling T^2^ statistics enables easily identifying outliers as those study samples located far-away the 95% Hotelling T^2^ ellipse in the PCA scores plot (Figure 3A) and those with extreme values in the T^2^ range plot (Figure 3B). Complementarily, observations showing high residual variance unexplained by the PCA model can be identified using the DmodX plot (Figure 3C). However, we recommend excluding potential outliers only in case that noticeable analytical or chemical anomalies in raw data could explain these behaviors, e.g., significantly different number of detected peaks, which could be indicative of human errors during sample preparation, technical issues during MS-based analysis (e.g., inefficient ionization), or sample contamination.

### Quality Markers for Addressing Preanalytical Factors

The preanalytical phase is well-recognized to be a major source of variability and errors, as collection and preprocessing of biological samples is frequently performed in clinical settings by staff with limited research experience. This is especially critical in multicenter and biobank-based studies, where samples are collected at different laboratories and over long-time periods. Accordingly, the implementation of SOPs for proper sampling and preprocessing is crucial to avoid contamination, degradation, and metabolic alteration of biological samples,^32,33^ thereby ensuring that subsequent metabolomics analysis provides an accurate reflection of the actual *in vivo* metabolic profile. This requires strict control of every step along the entire preanalytical phase, including sample collection, preprocessing (e.g., centrifugation), aliquoting, transport, storage, and thawing cycles. However, common strategies for QC assessment typically focus on addressing analytical quality,^13−16^ without considering the impact of preanalytical factors on metabolic integrity of samples under investigation.

Inappropriate quenching of biological samples may result in *ex vivo* metabolic reactions that are mediated by residual enzymatic activities (e.g., release of protease-derived peptides, hydrolysis of lipids).^34,35^ Furthermore, exposure to air and light has been associated with chemical transformations in readily oxidizable and labile metabolites.^36,37^ In blood samples, delayed processing and inadequate temperature during handling can overexpress anaerobic metabolism in erythrocytes and, consequently, alter circulating levels of energy-related metabolites.^37−39^ In this respect, hemolysis may also impact the serum/plasma metabolome as a result of the release of intracellular metabolites and the exacerbation of metabolic reactions triggered by erythroid enzymes.^40^ On the other hand, it has repeatedly been reported that urine samples are prone to suffer from profound metabolic alterations caused by bacterial overgrowth and chemical degradation.^41,42^ On this basis, we propose here a panel of metabolites known to be strongly influenced by the abovementioned preanalytical errors (Table 2), which can be monitored as markers of sample quality as a part of the QC*omics* protocol. The visualization of data in the form of box plots facilitates the detection of observations showing abnormal levels for these quality markers (i.e., peak intensities over ±3 × IQR), which could be indicative of improper handling/storage of the biological sample. As these metabolites can be influenced by a myriad of physiological and pathological stimuli, we recommend monitoring various of them before considering the exclusion of samples. Although this panel of markers has been designed for blood and urine, which are the most commonly employed biological matrices in metabolomics, this QC strategy can easily be adapted by the user to other tissue-specific metabolites.

**Table 2:** Panel of Quality Markers to Assess the Influence of Preanalytical Errors. Normal concentration ranges were obtained from the Human Metabolome Database. Arrows indicate the effect that preanalytical factors have been reported to elicit on metabolite expression (↑ increased levels, ↓ decreased levels).

**Marker**	**Concentration Range**	**Pre-Analytical Effect**		**Reference**
		**Blood**	**Urine**	
Hypoxanthine	Blood: 0.1–14.7 μM	↑ (enzymatic reactions)		(34,35,39)
Sphingosine 1-phosphate	Blood: 0.3–0.5 μM	↑ (enzymatic reactions)		(35)
Ascorbic acid	Blood: 11.0–171.0 μM; Urine: 1.7–22.7 μmol/mmol creatinine	↓ (oxidation)	↓ (oxidation)	(36,41)
Dopamine	Blood: < 0.13 nM	↓ (oxidation)		(36,37)
d-Glucose	Blood: 3.1–6.9 mM	↓ (anaerobic reactions)		(37,38)
Lactic acid	Blood: 6.8–11.1 μM; Urine: 3.4–12.6 μmol/mmol creatinine	↑ (anaerobic reactions)	↑ (anaerobic reactions)	(37,38,41,42)
Pyruvic acid	Blood: 10.0–141.0 μM	↑ (anaerobic reactions)		(38,39)
Creatine	Urine: 9.0–135.0 μmol/mmol creatinine		↑ (bacterial growth)	(41,42)
Acetic acid	Urine: 2.5–106.0 μmol/mmol creatinine		↑ (bacterial growth)	(41,42)
Succinic acid	Urine: 1.1–14.5 μmol/mmol creatinine		↑ (bacterial growth)	(42)

### Data Quality Assessment

**Figure 4:**
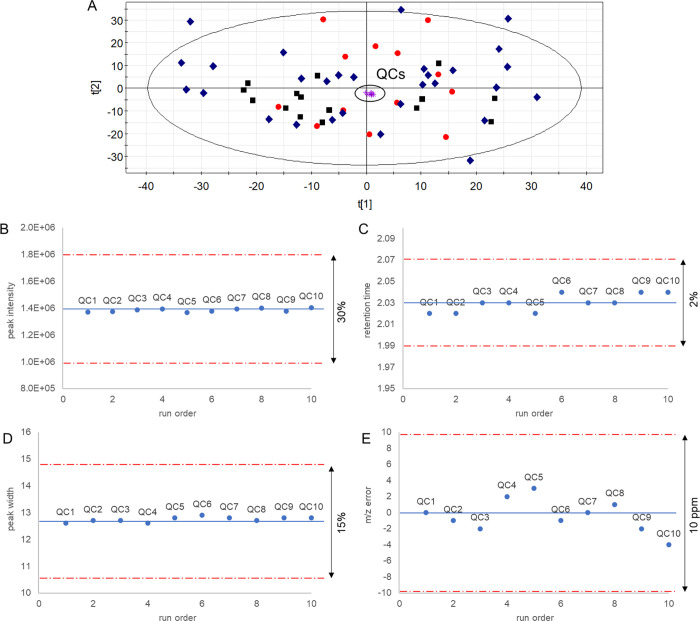
Data quality assessment. (A) Principal component analysis scores plot showing tight clustering of quality control samples; (B) time series plot with predefined tolerance windows for peak intensity; (C) time series plot with predefined tolerance windows for retention time; (D) time series plot with predefined tolerance windows for peak width; (E) time series plot with predefined tolerance windows for *m*/*z* error.

After data processing and cleaning as explained above (i.e., flagging background noise and carryover signals, monitoring drifts and “out-of-control” measurements, handling missing values and truly absent data, outlier detection, and removal of samples affected by preanalytical factors), the last step in QC*omics* involves evaluating overall data quality in terms of precision and accuracy. To this end, exploratory PCA can be first applied to the whole data set to confirm a tight clustering of QC samples in the scores plot as an indicator of system stability along the analytical run (Figure 4A). Then, method precision and accuracy must be estimated by applying the following acceptance criteria to chemical descriptors detected in QC samples: (i) RSD < 30% for peak intensity, (ii) RSD < 2% for RT, (iii) RSD < 15% for peak width, (iv) *m*/*z* error <10 ppm (this latter only for experiments conducted in high-resolution MS).^3,11^ Note that these acceptance criteria can be tailored according to the technical MS specifications. Additionally, time series plots with predefined tolerance windows can be used to visualize the reproducibility and stability of every quality metrics across the experiment (Figure 4B–E), thereby facilitating the identification of abnormal data points for potential exclusion or reanalysis. To conclude, the precision estimators can also be employed to discard peaks with a technical variation exceeding biological interindividual differences, as these molecular features are likely to introduce great variability in data and, consequently, hinder subsequent statistical modeling.

## Conclusions

The inherent complexity and variability of metabolomics data demand the application of robust QC strategies. However, despite numerous efforts made to increase awareness and promote best working practices among the metabolomics community, there is a considerable lack of standardization in QC workflows. To address this gap, we have developed a sequential multistep QC protocol, termed as QC*omics*, aimed to manage the most important challenges influencing data quality, including the correction of background noise and carryover, detection of gradual signal drifts and “out-of-control” measurements, dealing with missing values and truly absent data, detection and removal of outliers, monitoring of sample quality markers to address preanalytical errors, and overall data quality assessment. This tool generates easily interpretable outputs in the form of figures (e.g., Figures 2–4) and tables (e.g., tabulated RSD estimations), which could be annexed to scientific publications for consistent reporting of the data quality. Although it was initially designed for MS-based metabolomics, it should be noted that QC*omics* can also be used to manage other omics and MS data. In this sense, we would like to stress that some features of this QC protocol have successfully been applied in recent metabolomics and metallomics studies,^43−50^ which highlights its reliability to deal with complex and heterogeneous data. Therefore, we strongly believe that QC*omics* will facilitate the implementation of good QC practices, at both application and reporting levels, and thus become a gold standard in metabolomics research.
